# Night-to-Night Variability of Polysomnography-Derived Physiologic Endotypic Traits in Patients With Moderate to Severe OSA

**DOI:** 10.1016/j.chest.2022.12.029

**Published:** 2023-01-04

**Authors:** Christian Strassberger, Jan Hedner, Scott A. Sands, Thomas M. Tolbert, Luigi Taranto-Montemurro, Albert Marciniak, Ding Zou, Ludger Grote

**Affiliations:** a Center for Sleep and Vigilance Disorders, Institute of Medicine, Sahlgrenska Academy, University of Gothenburg, Gothenburg, Sweden; b Center for Sleep Medicine, Department of Respiratory Medicine, Sahlgrenska University Hospital, Gothenburg, Sweden; c Division of Pulmonary, Critical Care, and Sleep Medicine, Icahn School of Medicine at Mount Sinai, New York, NY; d Division of Sleep and Circadian Disorders, Brigham and Women's Hospital and Harvard Medical School, Boston, MA

**Keywords:** arousal threshold, collapsibility, endotypes, loop gain, muscle compensation, phenotypes, polysomnography, precision medicine, reliability, sleep-disordered breathing

## Abstract

**Background:**

Emerging data suggest that determination of physiologic endotypic traits (eg, loop gain) may enable precision medicine in OSA.

**Research Question:**

Does a single-night assessment of polysomnography-derived endotypic traits provide reliable estimates in moderate to severe OSA?

**Study Design and Methods:**

Two consecutive in-lab polysomnography tests from a clinical trial (n = 67; male, 69%; mean ± SD age, 61 ± 10 years; apnea-hypopnea index [AHI] 53 ± 22 events/h) were used for the reliability analysis. Endotypic traits, reflecting upper airway collapsibility (ventilation at eupneic drive [V_passive_]), upper airway dilator muscle tone (ventilation at the arousal threshold [V_active_]), loop gain (stability of ventilatory control, LG1), and arousal threshold (ArTh) were determined. Reliability was expressed as an intraclass correlation coefficient (ICC). Minimal detectable differences (MDDs) were computed to provide an estimate of maximum spontaneous variability. Further assessment across four repeated polysomnography tests was performed in a subcohort (n = 22).

**Results:**

Reliability of endotypic traits between the two consecutive nights was moderate to good (ICC: V_passive_ = 0.82, V_active_ = 0.76, LG1 = 0.72, ArTh = 0.83). Variability in AHI, but not in body position or in sleep stages, was associated with fluctuations in V_passive_ and V_active_ (*r* = –0.49 and *r* = –0.41, respectively; *P* < .001 for both). MDDs for single-night assessments were: V_passive_ = 22, V_active_ = 34, LG1 = 0.17, and ArTh = 21. Multiple assessments (mean of two nights, n = 22) further reduced MDDs by approximately 20% to 30%.

**Interpretation:**

Endotypic trait analysis using a single standard polysomnography shows acceptable reliability and reproducibility in patients with moderate to severe OSA. The reported MDDs of endotypic traits may facilitate the quantification of relevant changes and may guide future evaluation of interventions in OSA.

FOR EDITORIAL COMMENT, SEE PAGE 1016

**Take-home Points**

**Study Question:** Does a single-night assessment of polysomnography-derived endotypic traits provide reliable estimates in patients with OSA?

**Results:** Nine endotypic traits showed moderate to good reliability, both in the short-term and long-term. The analysis was extended further to provide minimal detectable differences (MDDs) as thresholds to evaluate changes beyond spontaneous variability.

**Interpretation:** Polysomnography-derived endotypic traits from a single-night assessment provide robust markers of the ventilatory control system and upper airway pathophysiologic features in OSA. MDDs of endotypic traits may be used to identify treatment responders and to guide clinical decisions in future clinical practice. The average of consecutive recordings can be used to reduce variance further.

OSA is characterized by repetitive upper airway (UA) collapse during sleep, concomitant intermittent hypoxia, arousals from sleep, increased risk for cardiovascular and metabolic comorbidities, and a compromised quality of life.^1^ However, the heterogeneity of clinical presentations cannot be captured by the traditional classification of disease severity based on frequency of respiratory disturbances.^2^ Pathophysiologic mechanisms, including high upper airway collapsibility, insufficient muscle compensation, ventilatory instability, and low arousal threshold have been described as potential underlying causes.^3^ Yet, standard methods to quantify these pathways require complex sleep study protocols, limiting clinical accessibility.

To overcome these limitations, an advanced modelling technique has been developed to estimate the key endotypic traits from routinely collected signals in clinical polysomnography.^4^, ^5^, ^6^ Several studies applied this approach to address responses to various OSA treatments,^7^^,^^8^ the potential to predict treatment success,^9^ or the effectiveness of therapeutic intervention in cohorts of mixed disease expressions.^10^ Using this method to investigate the pathophysiologic features of OSA in clinical practice may lead to recognition of clinical phenotypes and eventually may enable steps toward precision medicine and personalized treatment in this disorder.^11^

The use of endotypic traits for clinical classification of patients with OSA requires a high intraindividual stability and repeatability, which was investigated recently,^12^ whereas test-retest variability has not been quantified systematically. In fact, it is unclear to what extent these endotypic traits are influenced by known night-to-night variability of OSA severity and physiologic conditions during sleep.^13^ In the current study of moderate to severe sleep-disordered breathing, we explored night-to-night variability of endotypic traits and defined the thresholds for differences unlikely to result from spontaneous variability (minimal detectable difference [MDD]).

## Study Design and Methods

### Study Participants and Data Collection

**Figure 1:**
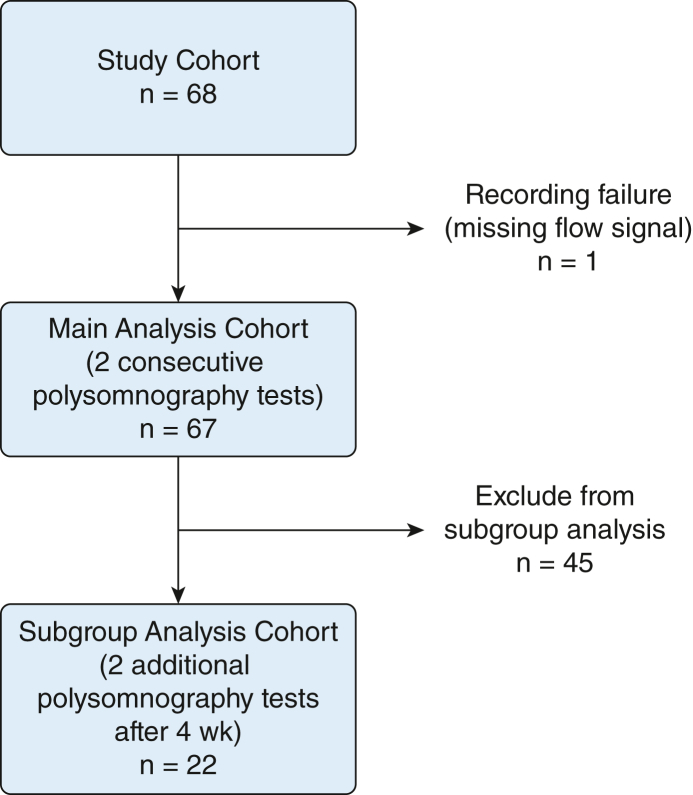
Study flow chart of the presented analysis. Main analysis was performed on 67 participants with two consecutive polysomnography tests for evaluation of night-to-night variability. A subgroup analysis was performed of 22 participants with a total of four repeated polysomnography tests available over the course of 4 weeks.

The data used for the present evaluation was obtained from a clinical trial described in detail elsewhere.^14^ In brief, this was a randomized, placebo-controlled safety and tolerability study evaluating a potential drug treatment for sleep apnea. Inclusion criteria were age 18 to 75 years, BMI ≥ 20 kg/m^2^ and ≤ 35 kg/m^2^, apnea-hypopnea index [AHI] ≥ 15 events/h, Epworth Sleepiness Scale score ≥ 6, and previous experience with CPAP, terminated because of nonacceptance, nontolerability, or both at least 4 weeks before the study. Patients (n = 68) underwent two consecutive standardized in-laboratory polysomnographic sleep recordings at baseline and an additional two nights after 4 weeks. Evaluation of night-to-night variability and test-retest reliability was performed on the two consecutive baseline assessments for all participants (main analysis cohort). Additional evaluation was performed using only the placebo-treated cohort (n = 22) to assess potential long-term variability after 4 weeks (subgroup analysis cohort). One participant was excluded because of lack of valid flow signal. A study flow chart is presented in Figure 1. The study was performed according to the tenets of the Declaration of Helsinki for clinical trials, and oral and written informed consent were obtained from each participant before entry into the study. The protocol was registered in the European Union Clinical Trials Register (Identifier: 2017-004767-13), and the current analysis was approved by the Swedish Ethical Review Authority (registration numbers 045-18 and 2020-06237).

### Sleep Study

Full-night, attended, in-laboratory polysomnography recordings (Embla A10 system; Flaga) were obtained in accordance with standards set by the American Academy of Sleep Medicine.^15^ The polysomnography recordings montage included EEG, electrooculography, chin and left and right anterior tibialis electromyography, and ECG. In addition, abdominal and thoracic respiratory effort belts, nasal pressure, nasal-oral thermistor, body position, and finger pulse oximetry were recorded.

All recordings were scored manually by a single sleep technologist in accordance with the American Academy of Sleep Medicine criteria.^15^ Arousal duration was determined carefully. Hypopneas were defined by the presence of a ≥ 3% desaturation, an arousal, or both. The frequency of apneic and hypopnic events was captured and calculated as the AHI. Desaturation events of ≥ 4% were used to determine the oxygen desaturation index (ODI). The derived sleep variables included total sleep time (TST), sleep efficiency, and sleep stages expressed as percentage of TST (eg, rapid eye movement [REM] percentage).

### Endotypic Traits Assessment

**Figure 2:**
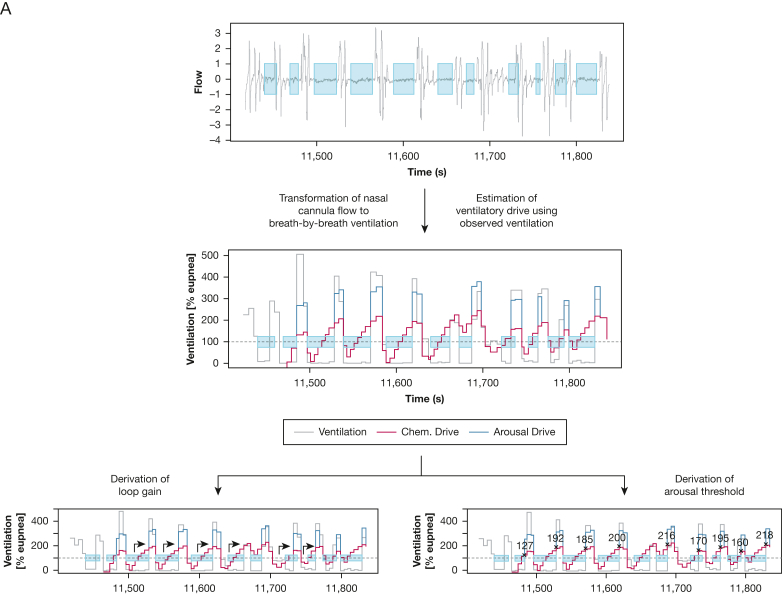

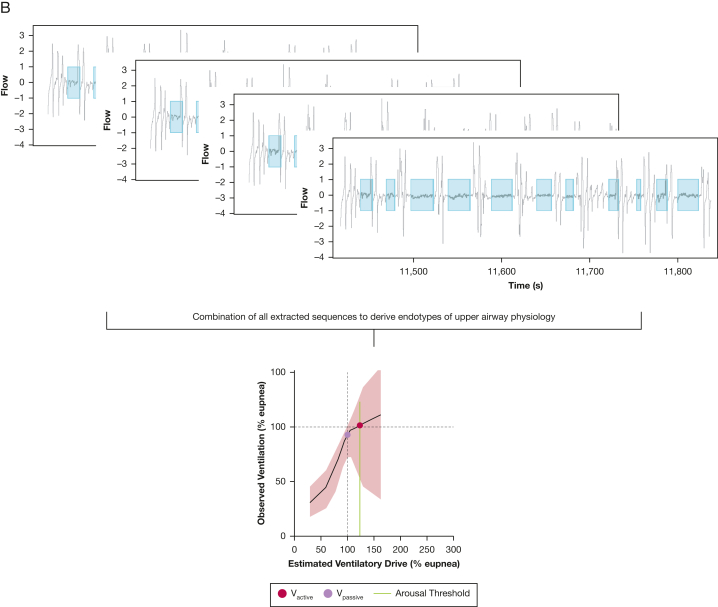
Conceptual visualization of the computation of endotypic traits. A, The ventilatory control system is evaluated by a breath-by-breath analysis of individual 7-min sequences of disturbed breathing. Parameters are derived based on the estimated ventilatory drive. B, Upper airway characteristics are evaluated based on a combination of all evaluated 7-min sequences. Parameters are derived based on the observed ventilation at different levels of ventilatory drive as a surrogate of upper airway muscle activity.

Endotypic traits were derived according to previously described methodology by Sands and colleagues^4^^,^^5^ and Terrill and colleagues^6^ using an automated analysis tool (PUPBeta, Phenotyping Using Polysomnography, version 02/2022). In brief, the nasal pressure signal was transformed to respiratory flow and used as input, together with manually scored apneas, hypopneas, and arousal events. The recording was split into windows of 7 min’s duration, which were used to derive the endotypic traits (Fig 2). Loop gain was computed as the response to both, a 1 cycle/minute disturbance (LG1) and at the natural frequency (LGn). Pharyngeal muscle activity was addressed by reporting the ventilation at minimal, and eupneic ventilatory drive (V_min_ and V_passive_), as well as at the ventilatory drive corresponding to the arousal threshold (V_active_). Endotypic traits were evaluated separately during REM and nonrapid eye movement (NREM) sleep and in the supine and nonsupine positions. Although endotypic traits were evaluated in both REM and NREM sleep, emphasis was placed on measurements from NREM sleep, because original validation studies were performed in this sleep stage. Nine separate endotypic traits were computed and used in the final evaluation, describing the ventilatory control system (LG1, LGn, arousal threshold, ventilatory response to arousals, and time delay) and UA pathophysiologic features (V_min_, V_active_, and V_passive_, and muscle compensation [V_comp_ = V_active_ – V_passive_]).

### Statistical Analysis

Data handling, visualization, and statistical analysis were performed using R version 4.1.3 software (R Foundation for Statistical Computing). Preprocessing was applied on endotypic traits V_active_ and V_passive_ to correct previously described floor and ceiling effects.^4^ In detail, a logit function, $\ln\left(\frac{x}{1-x}\right)$, was applied to both V_active_ and V_passive_, and then linearly back transformed for presentation within the scale (0,1). Similar corrections were reported previously.^16^ Additional information on the transformation of UA characteristics is described in e-Appendix 1 (e-Figs 1-4).

Statistical analysis was conducted independently on both analysis cohorts (n = 67 on consecutive nights and n = 22 within 4 weeks). Normal distribution was not assumed for parameter distribution on individual assessments, but was confirmed for the differences between measurements using Shapiro-Wilk normality test, together with visual inspection of distribution patterns and normal Q-Q plots. Descriptive data are presented as median (interquartile range) for all individual assessments. As a measure of agreement, differences between assessments are presented as mean ± SD and were evaluated using a paired *t* test. Because of the multiple comparisons, threshold to assume significant differences was set to 0.003 (Bonferroni adjustment). For the subgroup analysis cohort, long-term variability was determined by comparing the average of the two consecutive recordings at baseline to the average of the two consecutive recordings at 4 weeks to control for short-term, night-to-night variability. Intercorrelations have been investigated between the variation in phenotypic traits and standard polysomnography parameters. Visualization of variation between consecutive recordings was performed using Bland-Altman plots. Intraclass correlation coefficients (ICC; derived using the R package psych^17^) were reported as a measure of reliability and were evaluated according to recently published guidelines^18^ as a two-way mixed-effects model on absolute agreement. Reliability was interpreted as moderate (ICC > 0.5) or good (ICC > 0.75).

For evaluation of changes between consecutive recordings, a distribution-based method, the SEM, was applied. The SEM describes the statistical precision of a single measure, that is, with a confidence of 67% that the true value lies within a range of ± SEM. The MDD is derived based on the SEM. The MDD describes a threshold within which a repeated measure may fluctuate with high certainty, that is, 95%. Therefore, a difference between two measures that exceeds the MDD can be considered significantly higher than potential night-to-night variability.

In the present analysis, MDD was computed based on SEM (derived using the R package rel^19^) as $MDD=1.96\times \sqrt{2}\times SEM$, following the approach described by Fleiss and Kingman.^20^ Both the main analysis cohort (67 × 2 polysomnography tests) and subgroup analysis cohort (22 × 2 polysomnography tests) were used to derive MDD for single-night assessments. The subgroup analysis cohort (22 × 4 polysomnography tests) additionally was used to evaluate MDD between average scores of two recordings. Bootstrapping with *i* = 500 iterations has been applied to randomly select two pairs of the four assessments for each participant to compute SEM.

Because of previously reported differences in endotypic traits by sleep stage and position,^21^, ^22^, ^23^ evaluation was performed in view of different combinations of these sleep characteristics. To be in line with previous publications, the main focus of this study was on the analysis of phenotypic traits during NREM sleep, including all body positions. Additional analysis of endotypes with respect to body position and sleep stages have been performed to provide guidance on potential applications in the routine clinical setting of automatically derived endotypic traits. One analysis was performed to address the overall expression of phenotypic traits during the entire sleep period, whereas an additional sensitivity analysis was conducted including only NREM sleep with predominant supine body position. To ensure a reasonable level of data quality, a third analysis was performed where recordings with ≤ 2 h of sleep in the supine body position were excluded. To provide additional context on the implications of imposing more liberal or stricter conditions, the number of evaluated 7-min windows in each subcohort were reported, together with the number of evaluated participants.

## Results

Baseline characteristics of the main analysis cohort (n = 67 subjects), as well as the secondary analysis cohort (n = 22) are presented in Table 1. Patients were predominantly male, overweight, and had moderate to severe OSA. More than one-third had known but well-controlled cardiometabolic morbidities. To provide additional background information, baseline relationships between endotypic traits and selected sleep metrics are described in e-Appendix 2 (e-Figs 5, 6, e-Tables 1, 2).

**Table 1:** Baseline Characteristics of the Two Cohorts Used for Night-to-Night (Main Analysis) and Long-Term (Secondary Analysis) Variability

Variable	Main Analysis Cohort (n = 67)	Secondary Analysis Cohort (n = 22)
Male sex	46 (69)	17 (72)
Age, y	61 ± 10	61 ± 10
BMI, kg/m^2^	27.7 ± 3.2	29.1 ± 3.0
BP, mm Hg		
Systolic	137 ± 13	136 ± 12
Diastolic	83 ± 9	83 ± 8
Diabetes	4 (6)	2 (9)
Hypertension	23 (34)	6 (27)
Apnea index, events/h	30 ± 24	29 ± 23
Obstructive	27 ± 22	27 ± 22
Mixed	3 ± 5	2 ± 3
Central	1 ± 1	0 ± 1
Hypopnea index, events/h	25 ± 12	25 ± 13

Values are presented as No. (%) or mean ± SD.

Evaluation of conventional polysomnography parameters between consecutive nights is shown in Table 2. Approximately normally distributed differences were found for all parameters. AHI tended to be lower, whereas sleep efficiency, TST, and percentage of REM sleep were higher in the second recording. Both AHI and ODI showed good reliability (ICC, 0.83 and 0.82, respectively).

**Table 2:** Night-to-Night Variability of Endotypic Traits During NREM Sleep in All Positions

Variable	Polysomnography 1	Polysomnography 2	Difference	*P* Value^a^	ICC (95% CI)
No. of participants	67	67	. . .	. . .	. . .
No. of 7-min windows	86 ± 38	92 ± 37	5 ± 31	.165	0.65 (0.49-0.77)
Polysomnography					
AHI, events/h	52.3 (34.5 to 74.6)	50.5 (35.2-68.8)	–3.4 ± 13.8	.046	0.82 (0.72-0.88)
ODI, events/h	32.4 (14.4 to 53.4)	27 (15.2 to 47.1)	–0.3 ± 13.2	.844	0.83 (0.74-0.89)
TST, min	387 (311 to 408)	397 (361 to 433)	25.8 ± 55.6	< .001	0.53 (0.30-0.70)
Sleep efficiency, %	81.1 (64.9 to 85.1)	82.6 (74.5 to 89.8)	3.6 ± 14.5	.045	0.58 (0.39-0.72)
REM sleep, %	13.6 (9.4 to 18.8)	16.5 (13.0 to 21.6)	2.6 ± 6.6	.002	0.50 (0.29-0.67)
Supine position, %	44.0 (22.0 to 71.0)	48.3 (31.3 to 61.2)	0.3 ± 33.2	.949	0.39 (0.16-0.57)
Ventilatory control system					
LG1	0.53 (0.45 to 0.62)	0.54 (0.48 to 0.61)	0.01 ± 0.09	.451	0.72 (0.58-0.82)
ArTh	110 (105 to 119)	111 (105 to 123)	2.4 ± 10.3	.061	0.83 (0.74-0.89)
VRA	17.6 (11.1 to 28.5)	19.9 (12.0 to 37.5)	2.7 ± 10.6	.039	0.83 (0.73-0.89)
LGn	0.49 (0.42 to 0.56)	0.48 (0.44 to 0.55)	0.01 ± 0.08	.544	0.69 (0.54-0.80)
Delay	14.3 (12.9 to 16)	14.7 (12.5 to 15.8)	0.11 ± 2.16	.691	0.66 (0.50-0.78)
Upper airway pathophysiologic features					
V_passive_^b^	78.7 (68.8 to 84.5)	80.0 (71.2 to 86.0)	–0.1 ± 11.4	.956	0.82 (0.72-0.89)
V_active_^b^	96.9 (65.2 to 99.0)	95.7 (66.6 to 99.0)	0.1 ± 17.7	.982	0.76 (0.63-0.84)
V_comp_^c^	4.7 (–1.6 to 16.5)	6.3 (–0.9 to 12.9)	0.1 ± 11.7	.930	0.59 (0.40-0.72)
V_min_	42.6 (23.1 to 68.0)	50.1 (19.3 to 66.3)	0.5 ± 13.6	.748	0.87 (0.80-0.92)

Data are presented as median (interquartile range) or mean ± SD, unless otherwise indicated. AHI = apnea-hypopnea index; ArTh = arousal threshold (% eupnea); ICC = intraclass correlation coefficient; LGn = loop gain at natural frequency; LG1 = loop gain at 1 cycle/min; NREM = nonrapid eye movement; ODI = oxygen desaturation index; REM = rapid eye movement; TST = total sleep time; V_active_ = ventilation at the arousal threshold; V_comp_ = muscle compensation; V_min_ = ventilation at minimal ventilatory drive; V_passive_ = ventilation at eupneic drive; VRA = ventilatory response to arousals (% eupnea).

a Paired *t* test.

b Sigmoidal transformation applied.

c Derived using transformed V_active_ and V_passive_.

**Figure 3:**
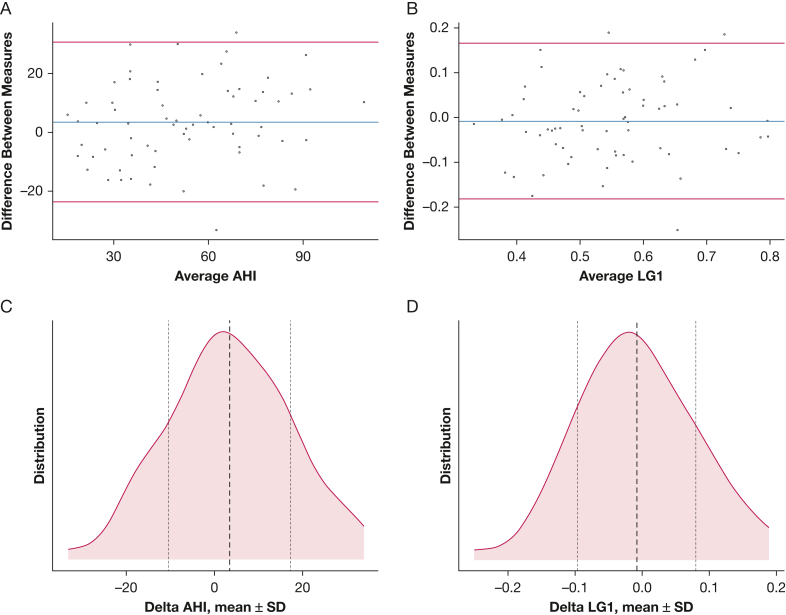
A-D, Bland-Altmann plots (A, B) and distribution of differences (C, D) for AHI and LG1 during nonrapid eye movement sleep. Shown data are taken from the main analysis cohort (n = 67), addressing the differences between two consecutive polysomnographic assessments. Distribution patterns show a normally distributed spread of differences, indicating no systematic bias in the tested parameters and justifying the use of mean results from consecutive recordings to reduce variability. AHI = apnea-hypopnea index; LG1 = loop gain at 1 cycle/min.

Results for the comparison of endotypic traits, derived during NREM sleep in all body positions, are included in Table 2. Approximately normally distributed differences were found for all traits. Using the paired *t* test, only ventilatory response to arousals showed a statistical trend toward increase on the second recording, but no significant differences were found after adjustment for multiple comparisons. Evaluating reliability by ICC showed moderate to good reliability for all endotypic traits, whereas V_comp_ and time delay showed the lowest reliability (ICC, 0.59 and 0.66, respectively). A visualization of variations between two consecutive nights in AHI and LG1 is shown in Figure 3. An in-depth evaluation of the correlation analysis is presented in e-Appendix 3 (e-Table 3). In summary, relevant correlation (ie, |*r*| > 0.4) between traditional polysomnography and novel endotype parameters was found for night-to-night changes in OSA severity (AHI and ODI) and night-to-night changes in upper airway characteristics (V_active_, V_passive_, and V_min_). The number of valid 7-min windows for evaluation of endotypic traits was higher in patients with severe compared with moderate sleep apnea. However, fluctuations in either the number of evaluated windows or in polysomnography parameters—such as TST, REM percentage, or time spent in the supine position—did not correlate with fluctuations in endotypic traits. Change in REM percentage was correlated with change in AHI.

An additional analysis of long-term variability was performed in the subgroup analysis cohort (n = 22 with four individual polysomnography tests over 4 weeks). None of the parameters showed significant differences between assessments and all parameters showed similar reliability compared with the previously reported night-to-night variability. However, the 95% CIs generally widened (Table 3).

**Table 3:** Long-term Variability of Tested Endotypic Traits During NREM Sleep in All Positions

Variable	Baseline	4 Wk	Difference	*P* Value^a^	ICC (95% CI)
No. of participants	22	22	. . .	. . .	. . .
No. of 7-min windows	89 ± 37	88 ± 37	–1 ± 33	.873	0.62 (0.26-0.82)
Polysomnography					
AHI, events/h	53.6 (35.4-68.9)	51 (32.3-69.1)	–3.0 ± 10.5	.191	0.89 (0.76-0.95)
ODI, events/h	37.3 (15.6-52.5)	36.9 (16.5-51)	–0.8 ± 10.7	.717	0.88 (0.74-0.95)
TST, min	392 (342-413)	380 (355-420)	6.5 ± 45.3	.507	0.63 (0.30-0.83)
Sleep efficiency, %	81.5 (70.6-85.5)	78.7 (73.6-87.7)	1.2 ± 9.3	.538	0.65 (0.33-0.84)
REM sleep, %	16.7 (12-22)	17.0 (12.1-21.4)	–0.8 ± 2.9	.229	0.89 (0.76-0.95)
Supine position, %	46.1 (29.2-53.7)	43.2 (24.9-53.9)	–4.5 ± 18.4	.270	0.70 (0.40-0.86)
Ventilatory control system					
LG1	0.56 (0.49-0.63)	0.53 (0.49-0.67)	0.00 ± 0.08	.851	0.72 (0.43-0.87)
ArTh	110 (105-120)	110 (105-123)	1.0 ± 7.6	.552	0.93 (0.84-0.97)
VRA	18.8 (8.5-31.0)	16.7 (11.2-24.8)	–0.5 ± 11.7	.838	0.69 (0.38-0.86)
LGn	0.48 (0.43-0.53)	0.47 (0.43-0.55)	0.00 ± 0.07	.823	0.63 (0.29-0.83)
Delay	13.5 (12.7-15.4)	14.0 (12.9-15.4)	0.2 ± 1.4	.545	0.87 (0.72-0.94)
Upper airway pathophysiologic features					
V_passive_^b^	81.8 (75.0-83.9)	82.3 (72.5-86.1)	–1.2 ± 7.2	.452	0.90 (0.77-0.96)
V_active_^b^	97.3 (72.9-99.0)	93.1 (63.1-99.0)	–2.8 ± 17.2	.445	0.74 (0.47-0.88)
V_comp_^c^	7.4 (1.5-14.8)	5.8 (-2.8-12.4)	–1.7 ± 13.1	.555	0.48 (0.08-0.75)
V_min_	43.4 (23.5-67)	46.5 (33.4-69.7)	2.9 ± 12.3	.282	0.88 (0.74-0.95)

Values are presented as median (interquartile range) or mean ± SD, unless otherwise indicated. AHI = apnea-hypopnea index; ArTh = arousal threshold (% eupnea); ICC = intraclass correlation coefficient; LGn = loop gain at natural frequency; LG1 = loop gain at 1 cycle/min; NREM = nonrapid eye movement; ODI = oxygen desaturation index; REM = rapid eye movement; TST = total sleep time; V_active_ = ventilation at the arousal threshold; V_comp_ = muscle compensation; V_min_ = ventilation at minimal ventilatory drive; V_passive_ = ventilation at eupneic drive; VRA = ventilatory response to arousals (% eupnea).

a Paired *t* test.

b Sigmoidal transformation applied.

c Derived using transformed V_active_ and V_passive_.

Reliability of endotypic traits in different subsets of the data defined by sleep stage and body position are presented in Table 4. Analyses have been performed using the main analysis cohort. The ICC showed moderate to good reliability between the two assessments for all variables. In general, a reduction in the number of participants or the number of 7-min windows was observed when stricter criteria were applied (Table 4), together with increases in variability and overall uncertainty. However, parameters describing UA characteristics (V_min_, V_active_, V_passive_, and V_comp_) showed improvements in the analysis only during supine positioning after assuring a minimum time spent in the supine position of > 2 h. Additional results on this sensitivity analysis are present in e-Appendix 4 (e-Tables 4-7)

**Table 4:** Reliability of Endotypic Traits During the Entire Sleep Period and Subdivided According to Sleep Stages and Body Positions

Variable	All Sleep, All Positions	NREM Sleep		
		All Positions	Supine Position	Supine Position > 120 min
No. of participants	67	67	52	34
No. of 7-min windows	128 ± 44	89 ± 37	54 ± 36	66 ± 36
Ventilatory control system				
LG1	0.75 (0.63-0.84)	0.72 (0.58-0.82)	0.52 (0.29-0.69)	0.57 (0.30-0.76)
ArTh	0.87 (0.79-0.92)	0.83 (0.74-0.89)	0.61 (0.40-0.75)	0.69 (0.47-0.83)
VRA	0.84 (0.74-0.90)	0.83 (0.73-0.89)	0.63 (0.44-0.77)	0.74 (0.55-0.86)
LGn	0.77 (0.65-0.85)	0.69 (0.54-0.80)	0.56 (0.34-0.72)	0.54 (0.25-0.74)
Delay	0.76 (0.64-0.85)	0.66 (0.50-0.78)	0.55 (0.32-0.71)	0.65 (0.41-0.81)
Upper airway pathophysiologic features				
V_passive_^a^	0.84 (0.75-0.90)	0.82 (0.72-0.89)	0.70 (0.54-0.82)	0.83 (0.68-0.91)
V_active_^a^	0.76 (0.63-0.84)	0.76 (0.63-0.84)	0.62 (0.42-0.76)	0.75 (0.56-0.87)
V_comp_^b^	0.56 (0.37-0.71)	0.59 (0.40-0.72)	0.26 (0.00-0.50)	0.55 (0.26-0.75)
V_min_	0.89 (0.83-0.93)	0.87 (0.80-0.92)	0.79 (0.66-0.87)	0.86 (0.73-0.93)

Values are presented as No., mean ± SD, or intraclass correlation coefficient (95% CI). ArTh = arousal threshold (% eupnea); LGn = loop gain at natural frequency; LG1 = loop gain at 1 cycle/min; NREM = nonrapid eye movement; V_active_ = ventilation at the arousal threshold; V_comp_ = muscle compensation; V_min_ = ventilation at minimal ventilatory drive; V_passive_ = ventilation at eupneic drive; VRA = ventilatory response to arousals (% eupnea).

a Sigmoidal transformation applied.

b Derived using transformed V_active_ and V_passive_.

SEM was derived and used to compute MDD for endotypic traits derived during NREM sleep. Both measures were derived to address changes between individual recordings, as well as between mean values of two recordings (subgroup analysis cohort). SEM with 95% CI, together with MDD, are presented in Table 5 for AHI, ODI, and the nine automatically derived endotypic traits.

**Table 5:** SEM and MDD for AHI, ODI, and Nine Automatically Derived Endotypic Traits

Variable	Individual Assessment, Main Cohort (n = 67 × 2)		Individual Assessment, Subgroup Analysis (n = 22 × 2)		Two-Night Average (n = 22 × 2)	
	SEM (95% CI)	MDD	SEM (95% CI)	MDD	SEM (95% CI)	MDD
AHI	10.0 (8.5-11.5)	27.7	9.4 (6.8-12.0)	26.1	7.5 (5.9-9.0)	20.7
ODI	9.3 (7.7-10.8)	25.7	9.2 (6.6-11.8)	25.5	6.5 (4.9-8.1)	18.0
LG1	0.06 (0.05-0.07)	0.17	0.06 (0.04-0.08)	0.17	0.05 (0.04-0.06)	0.14
ArTh	7.5 (6.1-8.8)	20.6	8.7 (5.9-11.6)	24.2	5.6 (4.2-6.9)	15.4
VRA	7.7 (6.3-9.0)	21.3	8.1 (5.5-10.7)	22.4	6.2 (3.8-8.6)	17.2
LGn	0.06 (0.05-0.06)	0.15	0.06 (0.04-0.08)	0.17	0.04 (0.03-0.05)	0.12
Delay	1.5 (1.2-1.8)	4.2	1.7 (1.1-2.4)	4.79	1.0 (0.8-1.3)	2.8
V_passive_^a^	8.0 (6.5-9.5)	22.1	10.3 (6.4-14.1)	28.5	5.5 (3.4-7.5)	15.1
V_active_^a^	12.4 (9.9-15.0)	34.4	14.6 (9.1-20.2)	40.5	9.5 (6.8-12.2)	26.4
V_comp_^b^	8.3 (6.8-9.7)	22.9	8.7 (6.0-11.5)	24.2	6.9 (4.1-9.7)	19.1
V_min_	9.6 (8.0-11.1)	26.5	7.6 (5.4-9.8)	21.0	6.8 (4.9-8.8)	19.0

Sixty-seven consecutive recordings were used to assess differences between individual recordings. Analysis was repeated with the subgroup analysis cohort to provide a context for comparison. Additionally, the 22 participants with four recordings each were evaluated to address differences in scores derived from averaging two nights to indicate the reduction of minimal detectable changes when applying a method to control for technical fluctuations. AHI = apnea-hypopnea index; ArTh = arousal threshold (% eupnea); LGn = loop gain at natural frequency; LG1 = loop gain at 1 cycle/min; MDD = minimal detectable difference; ODI = oxygen desaturation index; V_active_ = ventilation at the arousal threshold; V_comp_ = muscle compensation; V_min_ = ventilation at minimal ventilatory drive; V_passive_ = ventilation at eupneic drive; VRA = ventilatory response to arousals (% eupnea).

a Sigmoidal transformation applied.

b Derived using transformed V_active_ and V_passive_.

## Discussion

Our study demonstrated moderate to good agreement for nine endotypic trait variables assessed on consecutive nights, agreement that was maintained for at least a 4-week period. Night-to-night variability of endotypic traits was not associated with the fluctuations seen for OSA frequency measures, confirming independent stability in view of first-night effects. Results were extended further by contrasting influences of sleep stage and body position. Our study evaluated, for the first time to our knowledge, MDDs based on two consecutive individual assessments, as well as on the average of two recordings. The study provided insights into the clinical usefulness and potential limitations for derivation of OSA endotypes.

The concept of night-to-night variability in OSA was addressed in several previous studies.^24^^,^^25^ Our results, in general, confirmed previously described typical first night effects, such as a reduction of TST and the percentage of REM sleep.^26^ On a group level, AHI and ODI did not differ significantly on repeated assessments. However, we observed substantial variability in the AHI (SD, 13.8) in accordance with other studies on AHI variability in moderate to severe OSA based on clinical polysomnography (reported SDs of between 8 and 27).^27^, ^28^, ^29^, ^30^ Moreover, our estimate for reliability of the AHI (ICC, 0.82) is in line with previous analyses (ICC, 0.75-0.81).^31^, ^32^, ^33^ Correlation of nightly differences between AHI and time in the supine position have been reported^30^ that we were not able to reproduce, although we observed high variability in the supine position. Our study, investigating consecutive nights and variability over a span of up to 4 weeks, adds important information on previous evaluations of variability in polysomnography-derived endotypic trait measurements that evaluated variability within one night or changes over multiple years.^12^

No significant difference in endotypic trait variability was observed between consecutive recordings. Known physiologic relationships^3^ between OSA severity (AHI and ODI) and UA characteristics of collapsibility (V_active_, V_passive_, and V_min_) were confirmed (e-Fig 5, e-Table 1) and may explain the relationship between fluctuations in these characteristics. No other relevant correlations (ie, |*r*| > 0.4) were found, suggesting that the parameters mainly were independent of known physiologic fluctuations between nights. Importantly, we observed that both V_active_ and V_passive_ showed weaker agreement between nights at low numerical values (ie, high collapsibility). This, in turn, may influence the related measures of muscle compensation and may explain the increased uncertainty of this parameter. However, V_min_, which recently was proposed as a superior marker of collapsibility in a population-based study,^34^ generally was more stable and reliable for assessment of UA characteristics, as shown by higher values of ICC. Jointly, our findings suggest that pathophysiologic endotyping using a newly developed method indeed provides stable markers that may overcome between-night fluctuations and first-night phenomena.

In addition to the evaluation of night-to-night variability, this is the first study directly to address consequences of restricting the computation of endotypic traits to NREM sleep or supine body positioning. Previous studies^21^, ^22^, ^23^ demonstrated differences in pathophysiologic endotypes under these conditions that we were able to confirm in our dataset (e-Fig 6, e-Table 2). Despite this, the evaluation of the entire recording without subclassifying sleep stages and position lead to lower variance, as well as increased ICC. In fact, imposing stricter criteria evaluating only supine position and NREM sleep reduced agreement and reliability. With the exclusion of participants with a low duration (≤ 2 h) of supine sleep, we again found strong reliability in the UA characteristics, whereas the remaining parameters still showed weaker agreement. Our findings suggest that an important trade-off exist between technical model precision and physiologic stability. As a result, position-dependent analyses should be conducted only if sufficient data quality can be assured to reduce the estimate’s uncertainty. This could be realized by ensuring a certain data volume, which also was indicated in a related study by applying thresholds for a minimum number of evaluated 7-min windows.^12^ If technical limitations cannot be overcome, for example in ambulatory sleep assessments, it may be advisable to evaluate the entire night without separation of sleep and position, because this would provide more stable markers to identify phenotypic characteristics.

Our study provided a first suggestion for how to define minimal detectable changes in the proposed endotypic traits. Thresholds were determined by a distribution-based method widely used to quantify minimal detectable differences.^35^, ^36^, ^37^ We performed a traditional analysis using the two consecutive baseline assessments for deriving cutoffs for minimal detectable changes. Previous studies addressing MDD for OSA severity found generally lower AHI thresholds (MDD, 13-18 vs 28), but also were addressing less severe cohorts (mean AHI, 10-15 vs 53).^38^, ^39^, ^40^ A second analysis for calculation of MDD values was performed by evaluating the differences between the averages from two pairs of overnight polysomnography recordings, resulting in generally lower MDD thresholds. Future studies exploring these thresholds are warranted. Additional approaches, such as anchor-based methods,^41^ should be considered not only to investigate statistically detectable changes, but also to define clinically important differences related to outcome.

Several important study strengths should be recognized. Our study provided an in-depth investigation of the clinically important question on night-to-night variability and reproducibility of automatically derived endotypic traits using the PUPBeta software tool in a sizable cohort of patients with moderate to severe OSA. We demonstrated the stability of repetitive measures, gave a detailed evaluation on how various subanalyses affect the overall stability of the addressed measures and how these results can be placed into the context of clinical studies. The use of data collected from a clinical trial provides important insights into the application of a high-precision algorithm in the clinical setting. Our data strongly support the stability of derived endotypic traits for use in routine polysomnography assessments. Restricting analyses to specific sleep stage and position conditions may require stricter protocols to ensure reliable measurements.

The study also has important limitations. Our data were collected in a clinical trial and body posture was unrestricted. Consequently, some patients spent only a short time in the supine position, thereby reducing power and increasing variance in the statistical analysis of body position influences. After controlling for at least 2 h of supine positioning in the evaluated recordings, the cohort size reduced by around 50%. Controlling for body position increased the reliability of the UA characteristics, although without improving reliability in the remaining parameters. An important consequence of the data collection process was the evaluation of a preselected cohort: the study population comprised predominantly male, elderly patients with moderate to severe OSA and intolerance of CPAP therapy. This is a relevant cohort for the application of this methodology in the context of evaluating alternative treatment options in OSA, but differences in endotypic expressions with varying demographics have been reported previously.^42^, ^43^, ^44^ Furthermore, the initial study design allowed for an extensive evaluation of variability between consecutive recordings, but not for an in-depth evaluation of intraindividual variability or specific subtypes of OSA. Further studies are needed to determine if our findings can be generalized to other cohorts. This includes specifically addressing mild sleep apnea, strictly position-dependent OSA, REM-dependent OSA, OSA in women, and OSA in younger populations with predominantly UA abnormalities. Finally, it is important to bear in mind that this is a methodology requiring a full polysomnographic montage and good signal quality to enable the complex mathematical modelling. Although the validity has been studied extensively, the algorithmic realization and details, like transformation of UA characteristics, are updated and improved continuously. Currently, it is unclear to what extent physiologic and technical issues explain the observed variations. Additional evaluation in routine clinical settings is warranted to improve the methodology further.

## Interpretation

Endotypic traits derived from polysomnography tests using the PUPBeta software tool provided robust biomarkers over a span of up to 4 weeks. No direct relationship to spontaneous fluctuations in respiratory events was found, suggesting that endotypic traits reflect OSA mechanisms beyond momentary disease expressions. No systematic differences between assessments were observed, together with reasonable reliability assessed by ICC, indicating that a single night recording can be used for reliable endotypic characterization in clinical practice. Application of stricter criteria for sleep stages or body position not only may increase physiologic stability, but also may introduce statistical uncertainty. Detailed analyses on how to quantify the variation of novel endotypic traits with respect to different criteria on sleep and body position were performed. Moreover, this is the first study proposing thresholds to address statistically significant changes in endotypic traits beyond the natural course of night-to-night variation. Further studies applying this classification of endotypic traits in various clinical settings are warranted.

## Funding/Support

The clinical study that provided the data for the current analysis was sponsored by Destin GmbH, Hamburg, Germany (Carbonic anhydrase in sleep apnea [CAISA] study program), the Swedish Heart and Lung Foundation [Grants 20180585, 20210529, and 20210500], and from the Swedish state under the agreement between the Swedish government and the county councils [Grants LUA/ALF 966319 and 966283). C. S. is a PhD student financed by the 10.13039/501100005760University of Gothenburg, 10.13039/501100005761Sahlgrenska Academy [Grant PAR 2020/1175]. S.A.S. was funded by the National Heart Lung, and Blood Institute, 10.13039/100000002National Institutes of Health [Grant R01HL146697] and the 10.13039/100013981American Academy of Sleep Medicine Foundation [Grant 228-SR-20].

## Financial/Nonfinancial Disclosures

The authors have reported to *CHEST* the following: J. H. reports lecturing activities for Astra Zeneca, Bayer Pharma, and Itamar. He is also a collaborator in the European Union Hor020 and EUROSTAR programs (Sleep Revolution, APNEWAY). He is a co-owner of a licensed patent for sleep apnea treatment. L. G. reports lecturing activities for Resmed, Philips, Astra Zeneca, and Lundbeck as well as grant support for scientific projects from Bayer and the European Union Horizont 2020 and EUROSTAR programs (Sleep Revolution, APNEWAY). He is a co-owner of a licensed patent for sleep apnea treatment. S. A. S. served as a consultant to Nox Medical, Inspire, Merck, Apnimed, and Respicardia and received grant support from Apnimed, Prosomnus, and Dynaflex; his industry interactions are managed by the Brigham and Women’s Hospital. L. T.-M. is chief scientific officer of Apnimed and has financial interests in the same company outside the scope of the study; his interests are managed by the Brigham and Women’s Hospital. None declared (C. S., D. Z., T. M. T., A. M.).

## Supplementary Data

e-Online Data
