# Supplementary material for: Night-to-Night Variability of Polysomnography-Derived Physiologic Endotypic Traits in Patients With Moderate to Severe OSA
Source: Chest. 2023 Jan 4;163(5):1266–78. doi: 10.1016/j.chest.2022.12.029 (PMC10206510; doi:10.1016/j.chest.2022.12.029)
Supplement: e-Online Data [file mmc1.docx]

**Supplement Material**

**Night-to-night variability of PSG-derived physiological endotypic traits in patients with moderate to severe Obstructive Sleep Apnea**

**1. Transformation of Upper Airway Characteristics**

**Background**

Upper airway characteristics Vactive and Vpassive are derived by evaluating the observed ventilation vs. the modeled ventilatory drive^1^. These parameters are physiologically bounded between typical values of 0% and 100% of eupneic ventilation, leading to a floor and ceiling effect when compared with gold standard measures. To address this effect, a transformation of Vactive and Vpassive was proposed in previous publications, i.e., by

*“using a sigmoidal transformation function (slope of 1 at Vpassive = 50%) to handle the known floor and ceiling effects.” ^2^*

**Method**

In the current work, we realized this transformation by applying a logit function, i.e.,

$$\mathrm{logit}:\left( 0,1 \right)\mathbb{\to R;}logit\left( x \right) = \ln\left( \frac{x}{1-x} \right) .$$

Since values for Vactive and Vpassive can theoretically exceed the domain of (0,1), minimum and maximum constraints were applied as 0.01 ≤ x ≤ 0.99. For back-transformation, resulting values were shifted and linearly transformed with respect to the defined constraints to result within (0,1), i.e.,

$$\mathrm{logit}\left( x \right)\times\frac{0.49}{\mathrm{logit}\left( 0.99 \right)} + 0.5 \in\left( 0,1 \right).$$

Comparisons between raw and transformed Vpassive have been performed to visualize the implications of the applied transformation.

**Results and Discussion**

A visualization of Vpassive before and after transformation is shown in e-Figure 1. Most notably, this transformation is stretching the scale on high and low numerical values, giving a more detailed representation of the differences between subjects. This effect is additionally visualized using the distribution patterns of Vpassive before and after the transformation (e-Figure 2).

In addition, the transformed values of Vpassive showed a stronger linear relation between sleep apnea severity and collapsibility (e-Figure 3). Additional studies investigating the clinical significance of upper airway characteristics with or without the proposed transformation are warranted.

Investigation of night-to-night variability revealed a skewed distribution pattern of variability between consecutive recordings, i.e., lesser fluctuation for high numerical values. Since this transformation is rescaling high and low numerical values, the variation between consecutive recordings becomes evenly distributed, and overall variability is reduced (e-Figure 4).

Since we observed generally higher fluctuations in lower numerical values, it may be worth to consider applying a transformation, that is additionally shrinking the scale on low numerical values, for example $\ln\left( \frac{1}{1-x} \right)$, to further control for the uncertainty in lower numerical values. However, these considerations require additional studies aiming for finding appropriate transformation functions in relation to clinical applications, which is beyond the scope of the presented work.

**2. Baseline Characteristics of Endotypic Traits**

**Introduction and Methods**

To confirm the internal validity of the presented methodology and to highlight important relations between endotypic traits and OSA characteristics, additional baseline relations were investigated. These include the relation between increasing OSA severity, expressed by AHI. Moreover, the influence of sleep and position on these characteristics has been investigated by evaluating correlations with the REM sleep ratio and the ratio of supine position. Correlation analyses have been performed using spearman correlation. Group differences between REM- and NREM-derived endotypic traits have been tested using paired t-tests.

**Results and Discussion**

For visualization purposes, the evaluation of two functional, as well as anatomical endotypic characteristics, i.e., loop gain, arousal threshold, Vpassive, and Vmin, is presented in e-Figures 5 and 6. A complete overview of the performed analyses is presented in e-Tables 1 and 2. Higher AHI severity is generally associated with higher instability in the ventilatory control system (higher loop gain), increased arousal threshold, and ventilatory response to arousals, as well as higher levels of collapsibility (lower numerical values in Vpassive and Vmin).

Supine position and REM sleep ratio showed generally weaker relations with endotypic traits. Interestingly, traits describing upper airway collapsibility seem robust against increased proportions of supine sleep, despite known effects of supine posture on PCRIT^3^. It is however important to note, that the used cohort in this analysis is not representative for a detailed evaluation of either REM-dominant or position-dependent OSA. Therefore, additional studies are warranted to evaluate these relations in more detail.

**3. Correlation analysis of between-night fluctuations**

**Introduction and Methods**

Physiological fluctuations and night-to-night variability are well-known for conventional polysomnographic parameters, such as the apnea-hypopnea index (AHI) or the oxygen desaturation index (ODI). The derivation of the endotypic traits presented in this work was performed by evaluating the responses to naturally occurring upper airway obstructions. An investigation of whether fluctuations in the number of apneas and hypopneas are linked to fluctuations observed in endotypic traits is therefore warranted. We performed pairwise Spearman correlation analyses between conventional PSG parameters and endotypic traits. Relevant correlation was considered if the coefficient exceeds a threshold of 0.4. Investigated PSG parameters were AHI, ODI, total sleep time (TST, minutes), REM sleep time (% of TST), time spent in the supine position (minutes), and Supine AHI (events/hour). All parameters were investigated as absolute values (mean of two consecutive recordings), as well as their difference between the two recordings. Endotypic traits were analyzed with respect to their fluctuations between the two recordingsto investigate potential sources of night-to-night variability. We suspected endotypic trait fluctuations might be influenced by the number of evaluated 7-minute windows. Therefore, the absolute number of windows, as well as the change in the number of windows between the two recordings, was included in both groups of parameters.

**Results and Discussion**

Complete results of the correlation analysis are presented in e-Table 3. Interestingly, few correlation coefficients exceeded the threshold of 0.4, highlighted with bold characters in the table. We did not find evidence that fluctuations in conventional sleep parameters were predictive of fluctuations in endotypic traits. Correlations were found between the total number of valid 7-minute windows and the total value of AHI and ODI, which is expected due to the selection criteria of those windows in presence of respiratory events. Additionally, moderate correlations were also detected between changes in AHI/ODI and fluctuations in UA characteristics, while there was no indication that the remaining traits are affected in a systematic way. This suggests a high interdependency between parameters describing UA collapsibility and OSA severity, which may therefore reflect effects of OSA-related night-to-night variability.

**4. Sensitivity Analysis – Influence of sleep stage and position**

**Methods**

The main outcome of the presented study was the analysis of endotypic traits during NREM sleep in all body positions. Previous applications of the presented methodology have demonstrated differences of endotypic expressions in different physiological states. To evaluate the extent of their impact on variability, three types of sensitivity analyses have been performed. In the first analysis, no restrictions on sleep and position have been applied, representing the most lenient criteria. A second analysis has been conducted, including only NREM sleep predominantly in the supine position. Since our cohort showed a high fluctuation of sleep time in the supine position, a third analysis was performed, including only subjects with > 120 minutes of sleep in the supine position in both recordings to ensure sufficient data quality.

**Results and Discussion**

Complete results of the analyses are presented in e-Tables 4 – 7. No significant bias could be observed for any parameter between the first and second recording. In general, a reduction in the number of subjects or the number of 7-minute-windows was observed, reflective of increased technical limitations, when stricter criteria were applied. Consequently, increases in variability and overall uncertainty were observed, as shown by higher standard deviations and lower values of ICC. Only traits describing UA characteristics were able to overcome these limitations after ensuring sufficient sleep time in the supine position. Despite previous reported differences in endotypic traits between REM and NREM sleep and a significant first-night effect increasing the percentage share of REM sleep, the highest test re-test reliability was observed when including all sleep stages in the analysis. This indicates an important relation between technical stability and physiologically introduced variance, which varies across different traits. If technical limitations cannot be controlled by other methods, it may be necessary to apply the most lenient criteria to ensure reliable results. Studies investigating the clinical significance of this type of analysis are warranted.

**Tables and Figures**


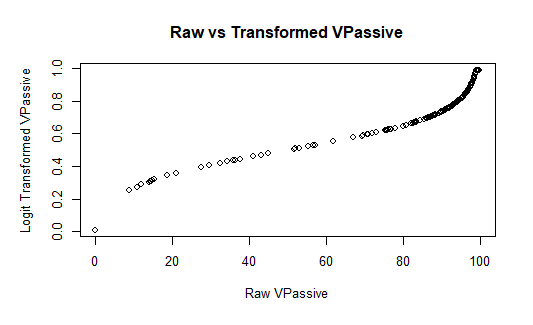


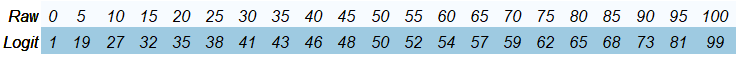


*e-Figure 1.* Visualization of raw versus logit-transformed Vpassive. Applying a sigmoidal transformation can be used for fine separation of high and low numerical values.


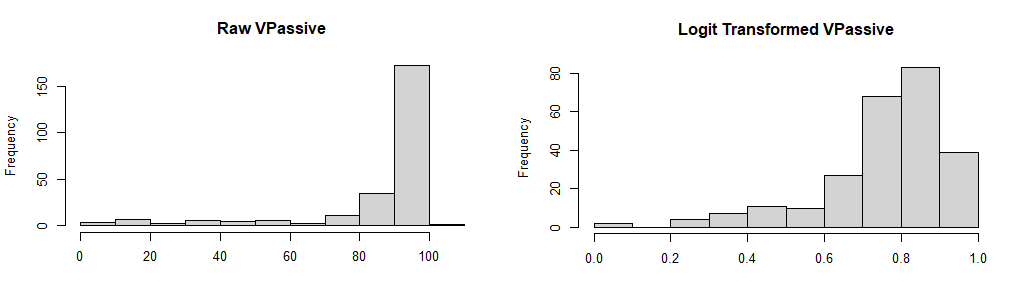


*e-Figure 2.* Visualization of distribution patterns of Vpassive before and after applying a logit transformation.


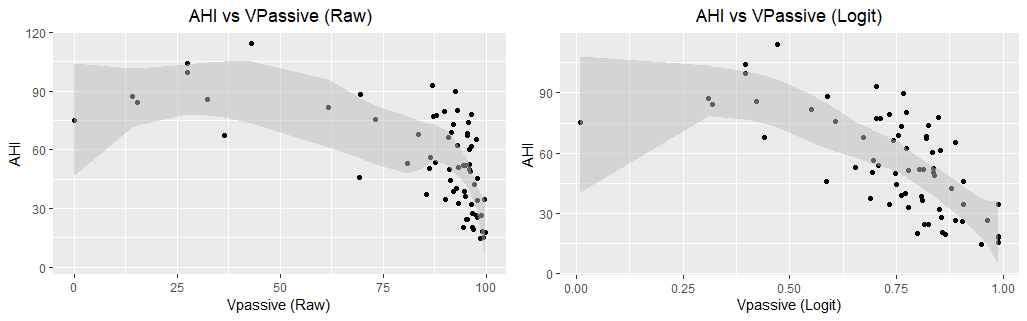


*e-Figure 3.* Relation between AHI and Vpassive, before and after applying a logit transformation. Stronger linear relation between the two parameters can be observed after logit-transforming Vpassive. Data taken from baseline recording PSG01. The shaded area is visualizing a local polynomial regression fit.


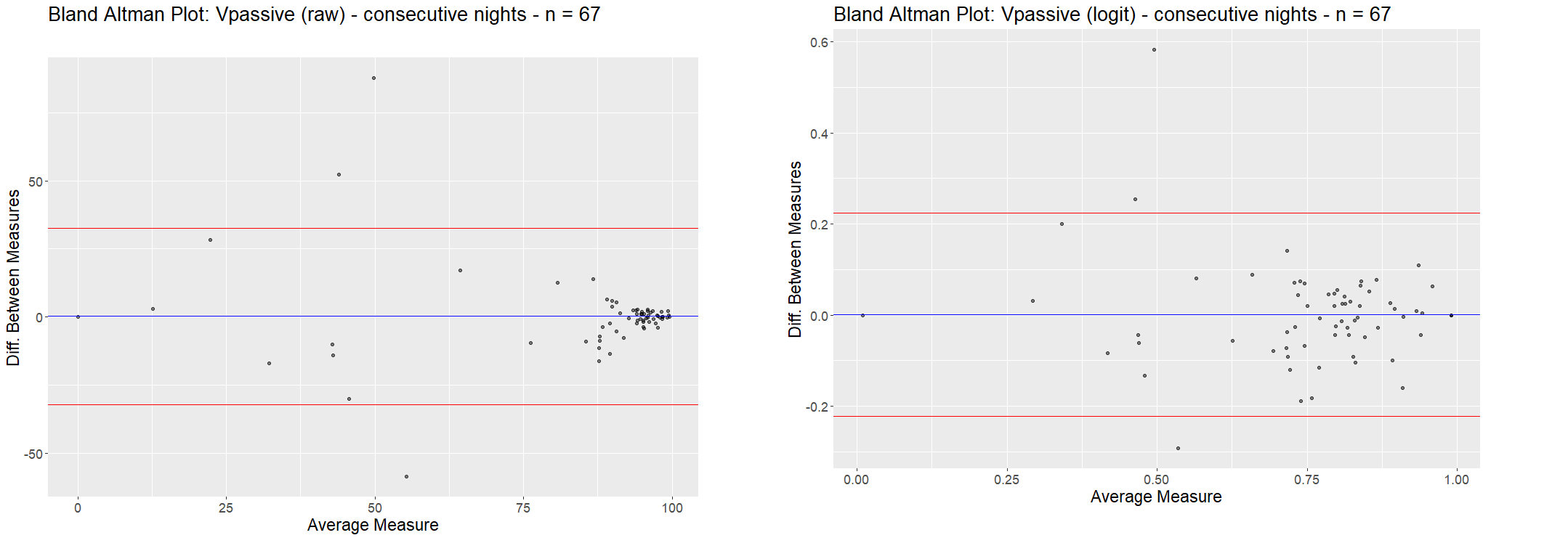


*e-Figure 4.* Bland Altman Plot, visualizing the night-to-night variability of Vpassive before and after applying a logit transformation. The initially skewed distribution pattern of differences between the two assessments can be partially addressed after applying the proposed transformation.


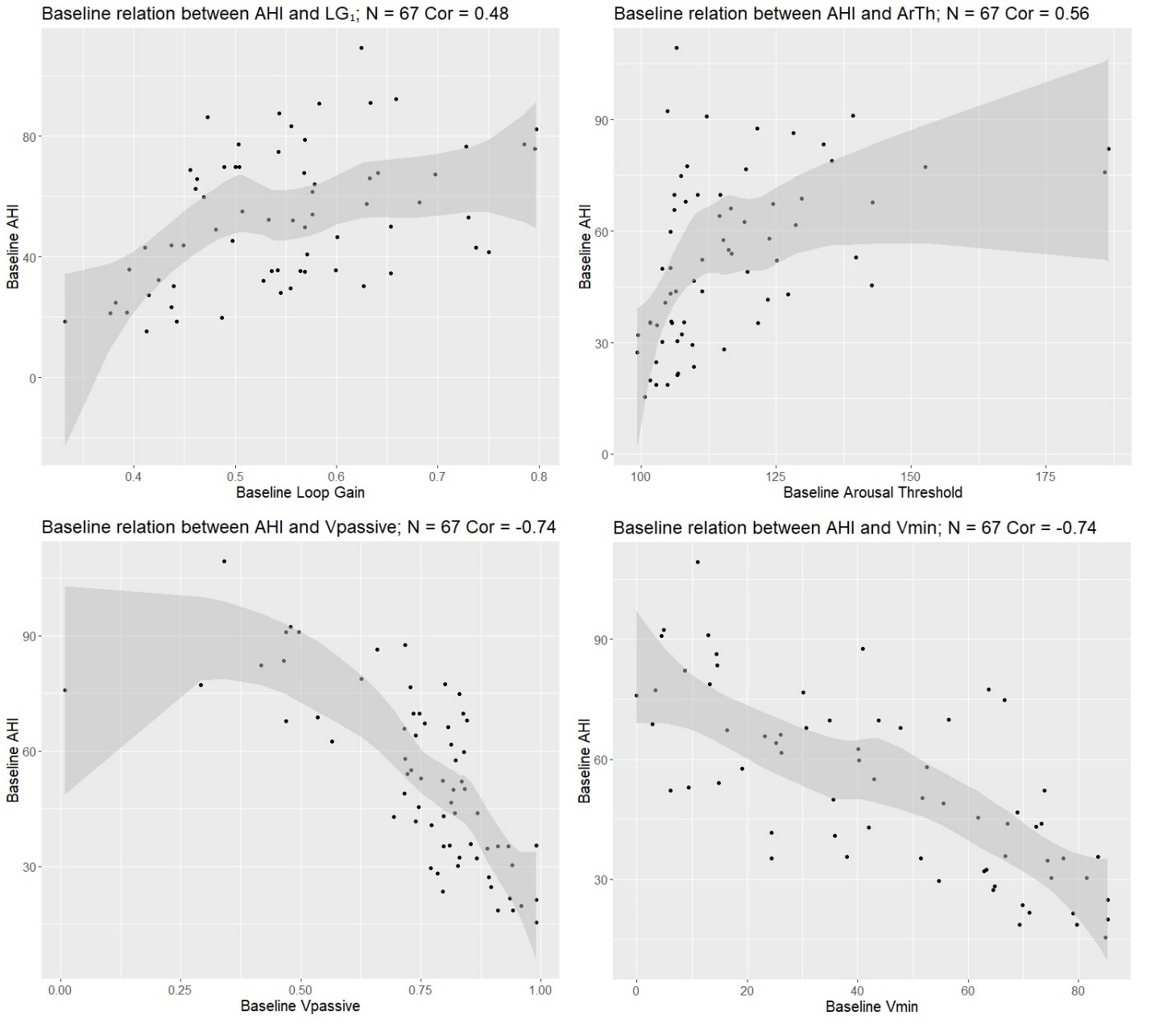


*e-Figure 5.* Relation between AHI and four endotypic traits, derived during NREM sleep, i.e., loop gain (LG1), Arousal Threshold (ArTh), Vpassive, and Vmin, quantified using spearman correlation coefficients (Cor). Depicted values are derived from the average of two baseline assessments. The shaded area is visualizing a local polynomial regression fit.


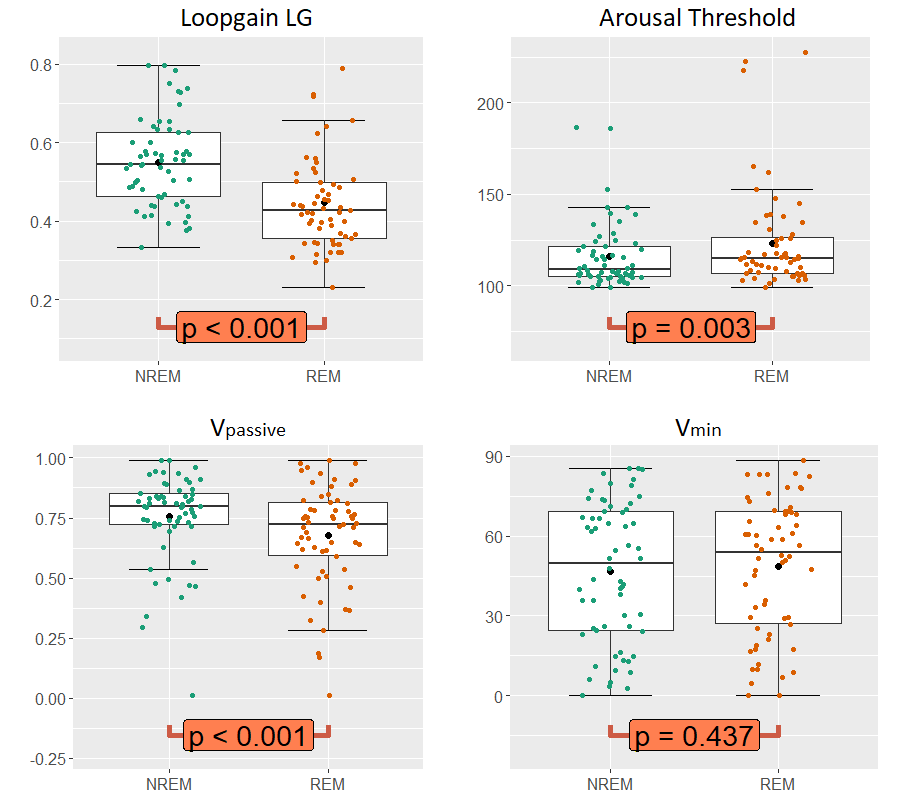


*e-Figure 6.* Differences between assessment of endotypic traits during REM and NREM sleep. Results shown for loop gain (LG1), Arousal Threshold, Vpassive, and Vmin, and compared using paired t-tests. Depicted values are derived from the average of two baseline assessments, individually evaluated for REM and NREM sleep in all body positions.

|  | LG1 | ArThres | VRA | LGn | delay | Vpassive^a^ | Vactive^a^ | Vcomp^b^ | Vmin |
| --- | --- | --- | --- | --- | --- | --- | --- | --- | --- |
| AHI | **0.48** | **0.56** | **0.50** | 0.30 | -0.22 | **-0.74** | **-0.58** | -0.18 | **-0.74** |
| Supine position (%) | 0.04 | 0.15 | **0.43** | -0.14 | -0.29 | -0.16 | -0.31 | **-0.40** | -0.17 |
| REM Sleep (%) | -0.04 | -0.35 | -0.18 | -0.06 | -0.06 | **0.45** | **0.44** | 0.30 | **0.40** |

e-Table 1: Pairwise Spearman correlation of parameters assessed during baseline. Endotypic traits are derived during NREM sleep in all body positions. LG1: loop gain at 1 cycle/minute, ArTh: arousal threshold (%eupnea), VRA: ventilatory response to arousals (%eupnea), LGn: loop gain at natural frequency, Vcomp: muscle compensation, Vactive/passive/min: Ventilation at different levels of muscle activity; ^a^sigmoidal transformation applied, ^b^derived using transformed Vactive and Vpassive.

| Endotypic Trait | REM (baseline median [IQR]) | NREM (baseline median [IQR]) | p-value |
| --- | --- | --- | --- |
| LG1 | 0.43 [0.36 – 0.50] | 0.55 [0.46 – 0.62] | p < 0.001 |
| ArThres | 115 [107 - 127] | 109 [105 - 121] | p = 0.003 |
| VRA | 22.3 [12.7 – 30.7] | 19.1 [11.7 – 31.6] | p = 0.557 |
| LGn | 0.45 [0.39 – 0.51] | 0.46 [0.42 – 0.55] | p = 0.005 |
| delay | 17.2 [14.9 – 18.6] | 14.6 [12.8 – 15.8] | p < 0.001 |
| Vpassive^a^ | 0.72 [0.60 – 0.81] | 0.80 [0.72 – 0.85] | p < 0.001 |
| Vactive^a^ | 0.80 [0.57 – 0.99] | 0.95 [0.67 – 0.99] | p = 0.002 |
| Vcomp^b^ | 0.04 [0.00 – 0.10] | 0.06 [0.00 – 0.14 ] | p = 0.748 |
| Vmin | 53.8 [27.3 – 69.1] | 49.6 [24.6 – 69.2] | p = 0.437 |

e-Table 2: Comparison of REM- and NREM-derived endotypic traits. Displayed mean values refer to the cohort mean of the derived endotypic trait at baseline. Significance was tested using a paired t-test. LG1: loop gain at 1 cycle/minute, ArTh: arousal threshold (%eupnea), VRA: ventilatory response to arousals (%eupnea), LGn: loop gain at natural frequency, Vcomp: muscle compensation, Vactive/passive/min: Ventilation at different levels of muscle activity; ^a^sigmoidal transformation applied, ^b^derived using transformed Vactive and Vpassive.

|  | All Sleep, All Position | | | | | | | | | | | |
| --- | --- | --- | --- | --- | --- | --- | --- | --- | --- | --- | --- | --- |
|  | 7-min W diff | 7-min W mean | AHI diff | LG1 diff | VRA diff | LGn diff | Delay diff | ArThres diff | Vpassive^a^ diff | Vactive^a^ diff | Vcomp^b^ diff | Vmin diff |
| AHI diff | 0.35 | -0.06 | **1** | 0.11 | 0.12 | 0.1 | -0.13 | 0.33 | **-0.62** | **-0.5** | -0.05 | **-0.48** |
| AHI mean | -0.04 | **0.58** | 0.2 | -0.06 | -0.09 | 0.03 | 0.05 | -0.15 | 0 | 0.07 | 0.13 | -0.05 |
| ODI diff | 0.35 | 0.07 | **0.66** | 0.14 | 0.27 | 0.18 | 0.01 | **0.56** | **-0.53** | **-0.48** | -0.12 | -0.39 |
| ODI mean | -0.04 | **0.66** | 0.16 | -0.03 | -0.11 | 0.06 | 0.07 | -0.07 | 0.03 | 0.08 | 0.13 | 0 |
| TST diff | **0.51** | 0.19 | -0.27 | 0.01 | 0.05 | -0.07 | -0.11 | -0.08 | 0.2 | 0.14 | -0.07 | 0.16 |
| TST mean | 0.05 | **0.54** | -0.33 | 0.12 | -0.2 | 0.08 | -0.03 | -0.06 | 0.11 | 0.09 | 0 | 0.25 |
| REM% diff | -0.14 | 0.28 | **-0.43** | -0.23 | 0.03 | -0.05 | 0.27 | -0.14 | 0.29 | 0.29 | 0.06 | 0.25 |
| REM% mean | 0.12 | 0.06 | -0.2 | 0.1 | -0.15 | 0.19 | 0.12 | -0.03 | 0.11 | 0.14 | 0.07 | 0.05 |
| Supine diff | 0.15 | 0.15 | 0.04 | -0.02 | -0.01 | -0.04 | 0.16 | 0.19 | -0.23 | -0.15 | -0.08 | -0.12 |
| Supine mean | 0.11 | **0.49** | -0.01 | 0.08 | -0.22 | 0.08 | -0.07 | -0.13 | 0.05 | 0.09 | 0.1 | 0.05 |
| SupAHI diff | 0.21 | -0.17 | **0.54** | -0.02 | 0 | 0.02 | -0.03 | 0.06 | -0.27 | -0.28 | -0.11 | -0.31 |
| SupAHI mean | -0.12 | 0.21 | 0.32 | -0.11 | 0.02 | -0.01 | 0.06 | -0.07 | -0.17 | -0.01 | 0.23 | -0.07 |
| 7-min W diff | **1** | 0.09 | 0.35 | 0.2 | 0.21 | 0.06 | -0.15 | 0.11 | -0.14 | -0.12 | -0.05 | -0.13 |
| 7-min W mean | 0.09 | **1** | -0.06 | 0.12 | -0.17 | 0.17 | 0.04 | -0.18 | 0.09 | 0.11 | 0.15 | 0.05 |
|  | NREM – All Position | | | | | | | | | | | |
| AHI diff | **0.42** | 0 | **1** | 0.06 | 0.1 | 0.05 | 0.01 | 0.28 | **-0.49** | **-0.41** | -0.08 | **-0.45** |
| AHI mean | -0.04 | **0.72** | 0.2 | 0.07 | -0.06 | 0.1 | 0.05 | -0.23 | 0.03 | 0.07 | 0.09 | -0.01 |
| ODI diff | **0.41** | 0.08 | **0.66** | 0.06 | 0.13 | 0.06 | 0.02 | **0.52** | **-0.45** | **-0.46** | -0.14 | -0.31 |
| ODI mean | -0.03 | **0.75** | 0.16 | 0.04 | -0.12 | 0.06 | 0.04 | -0.18 | 0.06 | 0.03 | 0.06 | 0.06 |
| TST diff | 0.37 | 0.11 | -0.27 | -0.02 | 0.13 | -0.15 | -0.09 | -0.06 | 0.18 | 0.05 | -0.18 | 0.22 |
| TST mean | 0.02 | 0.34 | -0.33 | 0.05 | -0.21 | 0.02 | 0.01 | -0.11 | 0.21 | -0.02 | -0.12 | 0.19 |
| REM% diff | **-0.43** | 0.22 | **-0.43** | -0.13 | 0.07 | -0.11 | 0.04 | -0.16 | 0.35 | 0.22 | 0.01 | 0.33 |
| REM% mean | 0.05 | -0.21 | -0.2 | 0.04 | -0.1 | 0.05 | 0.11 | 0.04 | 0.14 | 0.04 | -0.03 | 0.03 |
| Supine diff | 0.06 | 0.16 | 0.04 | 0.02 | 0.03 | -0.12 | 0.08 | 0.09 | -0.07 | -0.13 | -0.12 | -0.01 |
| Supine mean | 0.14 | 0.35 | -0.01 | 0.17 | -0.17 | 0.17 | 0.08 | -0.19 | 0.15 | 0.04 | -0.07 | 0 |
| SupAHI diff | 0.26 | -0.12 | **0.54** | -0.08 | 0.01 | 0.07 | 0.11 | 0.12 | -0.3 | -0.28 | -0.13 | -0.33 |
| SupAHI mean | -0.13 | **0.46** | 0.32 | -0.05 | 0.05 | 0.02 | 0.03 | -0.21 | -0.19 | -0.04 | 0.24 | -0.01 |
| 7-min W diff | **1** | 0.02 | **0.42** | 0.01 | 0.28 | -0.11 | 0.01 | 0.11 | -0.19 | -0.09 | -0.06 | -0.22 |
| 7-min W mean | 0.02 | **1** | 0 | 0.15 | -0.16 | 0.12 | -0.04 | -0.3 | 0.1 | 0.07 | 0.06 | 0.09 |
|  | NREM - Supine | | | | | | | | | | | |
| AHI diff | 0.22 | 0.03 | **1** | 0.15 | 0.02 | 0.13 | 0.21 | 0.17 | -0.12 | -0.16 | -0.14 | **-0.41** |
| AHI mean | 0.04 | **0.47** | 0.33 | 0.04 | -0.14 | 0.12 | 0.09 | -0.32 | 0.25 | 0.28 | 0.16 | 0.06 |
| ODI diff | 0.34 | -0.02 | **0.69** | 0.23 | 0.14 | 0.19 | 0.02 | **0.41** | -0.26 | -0.23 | -0.07 | **-0.41** |
| ODI mean | 0.08 | **0.56** | 0.25 | 0.02 | -0.06 | 0.14 | 0.09 | -0.29 | 0.21 | 0.21 | 0.09 | 0.09 |
| TST diff | 0.23 | 0.18 | -0.25 | 0.25 | 0.27 | 0.16 | -0.05 | -0.1 | -0.08 | -0.02 | 0.03 | 0.07 |
| TST mean | 0.05 | 0.38 | -0.28 | 0.21 | -0.18 | 0.21 | -0.07 | 0.04 | -0.02 | -0.05 | -0.01 | 0.17 |
| REM% diff | -0.19 | 0.28 | **-0.45** | -0.02 | -0.04 | -0.01 | 0.01 | -0.2 | 0.3 | 0.32 | 0.23 | **0.41** |
| REM% mean | 0.06 | -0.06 | -0.2 | 0.25 | -0.06 | 0.24 | -0.01 | 0.25 | 0.01 | -0.01 | 0.01 | 0.02 |
| Supine diff | **0.73** | 0.22 | 0.06 | 0.29 | 0.06 | 0.17 | 0.07 | 0.11 | 0.07 | 0.11 | 0.12 | 0.01 |
| Supine mean | 0.16 | **0.76** | 0.06 | 0.35 | -0.09 | 0.36 | 0 | -0.05 | -0.09 | -0.02 | 0.02 | -0.01 |
| SupAHI diff | 0.15 | -0.14 | **0.61** | 0.15 | 0.16 | 0 | 0.03 | 0.17 | **-0.43** | -0.29 | 0.02 | **-0.47** |
| SupAHI mean | 0.03 | 0.25 | 0.29 | -0.12 | -0.09 | -0.07 | 0.15 | -0.31 | 0.33 | 0.34 | 0.2 | 0.11 |
| 7-min W diff | **1** | 0.1 | 0.22 | 0.32 | 0.21 | 0.22 | 0 | 0.2 | -0.14 | -0.01 | 0.11 | -0.26 |
| 7-min W mean | 0.1 | **1** | 0.03 | 0.23 | -0.23 | 0.26 | -0.05 | -0.27 | 0.12 | 0.21 | 0.18 | 0.12 |
|  | NREM – Supine > 120 minutes | | | | | | | | | | | |
| AHI diff | 0.26 | 0.11 | **1** | 0.07 | -0.07 | 0.02 | 0.22 | 0.16 | -0.01 | -0.1 | -0.14 | **-0.48** |
| AHI mean | 0.14 | **0.55** | 0.19 | -0.13 | -0.08 | 0.04 | 0.19 | -0.26 | 0.18 | 0.24 | 0.23 | 0.01 |
| ODI diff | 0.22 | 0.07 | **0.7** | 0.25 | -0.01 | 0.16 | -0.08 | **0.48** | -0.04 | -0.05 | 0.07 | -0.33 |
| ODI mean | 0.08 | **0.66** | 0.14 | -0.14 | -0.03 | 0.09 | 0.19 | -0.21 | 0.14 | 0.18 | 0.18 | 0.06 |
| TST diff | 0.18 | 0.16 | -0.28 | -0.07 | 0.19 | -0.12 | -0.13 | -0.09 | 0.08 | 0.14 | 0.15 | 0.31 |
| TST mean | -0.04 | 0.26 | -0.27 | 0.1 | -0.26 | 0.16 | -0.04 | 0.03 | 0.08 | -0.03 | -0.06 | 0.35 |
| REM% diff | -0.17 | 0.32 | **-0.46** | -0.06 | -0.05 | 0.19 | 0.26 | -0.14 | 0.35 | 0.32 | 0.12 | **0.52** |
| REM% mean | 0.04 | -0.15 | -0.04 | 0.38 | -0.2 | 0.35 | 0 | 0.32 | 0.17 | -0.01 | -0.05 | 0.15 |
| Supine diff | **0.66** | 0.24 | 0.07 | 0.26 | 0.04 | 0.13 | 0.13 | 0.24 | 0.14 | 0.1 | 0.09 | 0.15 |
| Supine mean | -0.08 | **0.63** | 0.02 | 0.12 | -0.14 | 0.36 | 0.21 | 0.03 | -0.09 | -0.22 | -0.23 | 0.07 |
| SupAHI diff | 0.24 | -0.11 | **0.68** | 0.13 | 0.12 | 0.07 | 0.14 | 0.17 | **-0.43** | -0.24 | 0 | **-0.69** |
| SupAHI mean | 0.1 | **0.55** | 0.23 | -0.21 | -0.16 | -0.08 | 0.16 | -0.29 | 0.25 | 0.34 | 0.29 | 0.11 |
| 7-min W diff | **1** | 0.13 | 0.26 | 0.36 | 0.09 | 0.22 | -0.11 | 0.36 | -0.06 | 0 | 0.09 | -0.16 |
| 7-min W mean | 0.13 | **1** | 0.11 | -0.05 | -0.23 | 0.21 | 0.15 | -0.18 | -0.03 | -0.01 | 0.05 | 0.1 |

e-Table 3: Pairwise Spearman correlation between conventional PSG parameters and novel endotypic traits. Correlations with |r| > 0.4 are printed in bold text. To address potential causes for variability in endotypic traits, differences between consecutive PSGs have been used as input, while PSG parameters have been used additionally as absolute value (mean of two PSGs). AHI: apnea-hypopnea index, ODI: oxygen-desaturation index, TST: total sleep time, REM: rapid eye movement sleep, SupAHI: AHI in supine position, 7-min W: Number of 7-minute windows for evaluation of endotypic traits, LG1: loop gain at 1 cycle/minute, ArTh: arousal threshold (%eupnea), VRA: ventilatory response to arousals (%eupnea), LGn: loop gain at natural frequency, Vcomp: muscle compensation, Vactive/passive/min: Ventilation at different levels of muscle activity; ^a^sigmoidal transformation applied, ^b^derived using transformed Vactive and Vpassive.

|  |  | PSG01 | PSG02 | Mean Difference ± SD | Paired t-test | ICC (95% CI) |
| --- | --- | --- | --- | --- | --- | --- |
|  | n Subjects | 67 | 67 |  |  |  |
|  | n 7-min Windows | 122 ± 45 | 134 ± 42 | 11 ± 33 | 0.007 | 0.69 (0.53 – 0.8) |
| Ventilatory Control System | LG1 | 0.52 [0.43 - 0.59] | 0.50 [0.46 - 0.59] | 0.00 ± 0.08 | 0.679 | 0.75 (0.63 – 0.84) |
|  | ArThres | 110 [106 - 121] | 113 [106 - 123] | 2.2 ± 9.3 | 0.060 | 0.87 (0.79 – 0.92) |
|  | VRA | 17.2 [10.2 - 29.4] | 18.9 [11.5 - 33.9] | 2.7 ± 10.8 | 0.042 | 0.84 (0.74 – 0.9) |
|  | LGn | 0.45 [0.40 - 0.57] | 0.48 [0.43 - 0.54] | 0.00 ± 0.06 | 0.685 | 0.77 (0.65 – 0.85) |
|  | delay | 14.8 [13.1 - 16.6] | 15.2 [14.1 - 16.2] | 0.19 ± 1.58 | 0.323 | 0.76 (0.64 – 0.85) |
| Upper Airway Pathophysiology | Vpassive^a^ | 77.9 [70.0 – 85.0] | 79.7 [70.5 – 84.7] | -0.6 ± 10.7 | 0.674 | 0.84 (0.75 – 0.9) |
|  | Vactive^a^ | 94.1 [63.3 – 99.0] | 96.6 [57.6 – 99.0] | -0.9 ± 17.9 | 0.695 | 0.76 (0.63 – 0.84) |
|  | Vcomp^b^ | 6.2 [-3.0 – 15.4] | 7.6 [-2.4 – 14.3] | -0.3 ± 12.0 | 0.831 | 0.56 (0.37 – 0.71) |
|  | Vmin | 48.0 [21.5 – 67.0] | 48.8 [18.9 – 64.8] | -0.7 ± 12.1 | 0.640 | 0.89 (0.83 – 0.93) |

*e-Table 4*: Night-to-night variability for endotypic traits assessed during the entire night in all position

|  |  | PSG01 | PSG02 | Mean Difference ± SD | Paired t-test | ICC (95% CI) |
| --- | --- | --- | --- | --- | --- | --- |
|  | n Subjects | 67 | 67 |  |  |  |
|  | n 7-min Windows | 86 ± 38 | 92 ± 37 | 5 ± 31 | 0.165 | 0.65 (0.49 – 0.77) |
| Ventilatory Control System | LG1 | 0.53 [0.45 - 0.62] | 0.54 [0.48 - 0.61] | 0.01 ± 0.09 | 0.451 | 0.72 (0.58 – 0.82) |
|  | ArThres | 110 [105 - 119] | 111 [105 - 123] | 2.4 ± 10.3 | 0.061 | 0.83 (0.74 – 0.89) |
|  | VRA | 17.6 [11.1 - 28.5] | 19.9 [12.0 - 37.5] | 2.7 ± 10.6 | 0.039 | 0.83 (0.73 – 0.89) |
|  | LGn | 0.49 [0.42 - 0.56] | 0.48 [0.44 - 0.55] | 0.01 ± 0.08 | 0.544 | 0.69 (0.54 – 0.8) |
|  | delay | 14.3 [12.9 - 16] | 14.7 [12.5 - 15.8] | 0.11 ± 2.16 | 0.691 | 0.66 (0.5 – 0.78) |
| Upper Airway Pathophysiology | Vpassive^a^ | 78.7 [68.8 – 84.5] | 80.0 [71.2 – 86.0] | -0.1 ± 11.4 | 0.956 | 0.82 (0.72 – 0.89) |
|  | Vactive^a^ | 96.9 [65.2 – 99.0] | 95.7 [66.6 – 99.0] | 0.1 ± 17.7 | 0.982 | 0.76 (0.63 – 0.84) |
|  | Vcomp^b^ | 4.7 [-1.6 – 16.5] | 6.3 [-0.9 – 12.9] | 0.1 ± 11.7 | 0.930 | 0.59 (0.4 – 0.72) |
|  | Vmin | 42.6 [23.1 – 68.0] | 50.1 [19.3 - 66.3] | 0.5 ± 13.6 | 0.748 | 0.87 (0.8 – 0.92) |

*e-Table 5:* Night-to-night variability for endotypic traits assessed during NREM sleep in all position

|  |  | PSG01 | PSG02 | Mean Difference ± SD | Paired t-test | ICC (95% CI) |
| --- | --- | --- | --- | --- | --- | --- |
|  | n Subjects | 52 | 52 |  |  |  |
|  | n 7-min Windows | 53 ± 39 | 55 ± 33 | 2 ± 36 | 0.645 | 0.52 (0.29 – 0.69) |
| Ventilatory Control System | LG1 | 0.53 [0.46 - 0.62] | 0.54 [0.46 - 0.62] | 0.00 ± 0.12 | 0.899 | 0.52 (0.29 – 0.69) |
|  | ArThres | 114 [105 - 126] | 113 [107 - 132] | 4.0 ± 17.8 | 0.109 | 0.61 (0.4 – 0.75) |
|  | VRA | 26.8 [13.9 - 38.7] | 26.5 [15.8 - 46] | 2.1 ± 18.9 | 0.422 | 0.63 (0.44 – 0.77) |
|  | LGn | 0.45 [0.39 - 0.56] | 0.47 [0.41 - 0.53] | 0.01 ± 0.10 | 0.534 | 0.56 (0.34 – 0.72) |
|  | delay | 13.6 [12.4 - 15.4] | 14.2 [12.3 - 15.7] | 0.28 ± 2.38 | 0.395 | 0.55 (0.32 – 0.71) |
| Upper Airway Pathophysiology | Vpassive^a^ | 71.2 [59.5 – 81.9] | 71.4 [50.7 – 85.6] | -1.3 ± 17.7 | 0.612 | 0.70 (0.54 – 0.82) |
|  | Vactive^a^ | 66.2 [45.9 – 99.0] | 68.6 [45 – 99.0] | 0.2 ± 24.5 | 0.952 | 0.62 (0.42 – 0.76) |
|  | Vcomp^b^ | 0.2 [-10.1 – 9.27] | 0.6 [-5.4 – 8.3] | 1.5 ± 16.7 | 0.533 | 0.26 (0 – 0.5) |
|  | Vmin | 42.0 [21.8 - 61.5] | 42.8 [14 – 63.9] | -0.4 ± 18.0 | 0.886 | 0.79 (0.66 – 0.87) |

*e-Table 6:* Night-to-night variability for endotypic traits assessed during NREM sleep in supine position

|  |  | PSG01 | PSG02 | Mean Difference ± SD | Paired t-test | ICC (95% CI) |
| --- | --- | --- | --- | --- | --- | --- |
|  | n Subjects | 34 | 34 |  |  |  |
|  | n 7-min Windows | 69 ± 38 | 62 ± 34 | -8 ± 34 | 0.183 | 0.54 (0.26 – 0.74) |
| Ventilatory Control System | LG1 | 0.55 [0.48 – 0.62] | 0.52 [0.45 – 0.60] | -0.03 ± 0.11 | 0.112 | 0.57 (0.3 – 0.76) |
|  | ArThres | 112 [105 - 127] | 113 [109 - 139] | 5.3 ± 17.5 | 0.087 | 0.69 (0.47 – 0.83) |
|  | VRA | 28.0 [13.1 - 47.4] | 28.7 [13.5 - 53.7] | 3.4 ± 16.8 | 0.241 | 0.74 (0.55 – 0.86) |
|  | LGn | 0.45 [0.39 - 0.56] | 0.45 [0.41 - 0.52] | -0.01 ± 0.10 | 0.442 | 0.54 (0.25 – 0.74) |
|  | delay | 13.5 [12.2 - 15.3] | 14.4 [12.3 - 15.5] | 0.43 ± 1.87 | 0.185 | 0.65 (0.41 – 0.81) |
| Upper Airway Pathophysiology | Vpassive^a^ | 75.4 [60.1 – 86.9] | 71.7 [51.9 – 85.8] | -1.0 ± 14.6 | 0.685 | 0.83 (0.68 – 0.91) |
|  | Vactive^a^ | 70.1 [45.7 – 99.0] | 70.1 [44.6 – 99.0] | -1.1 ± 21.1 | 0.761 | 0.75 (0.56 – 0.87) |
|  | Vcomp^b^ | 0.0 [-7.7 – 9.69] | 0.3 [-5.8 – 5.7] | -0.1 ± 13.1 | 0.971 | 0.55 (0.26 – 0.75) |
|  | Vmin | 37.9 [21.1 - 67.3] | 44.8 [14.4 - 67.4] | 0.6 ± 15.9 | 0.816 | 0.86 (0.73 – 0.93) |

*e-Table 7:* Night-to-night variability for endotypic traits assessed during NREM sleep in supine position with time spend in supine position > 120 min

CI: confidence interval, LG1: loop gain at 1 cycle/minute, ArTh: arousal threshold (%eupnea), VRA: ventilatory response to arousals (%eupnea), LGn: loop gain at natural frequency, Vcomp: muscle compensation, Vactive/passive/min: Ventilation at different levels of muscle activity; ^a^sigmoidal transformation applied, ^b^derived using transformed Vactive and Vpassive.

**References**

1. Sands SA, Edwards BA, Terrill PI, et al. Phenotyping Pharyngeal Pathophysiology using Polysomnography in Patients with Obstructive Sleep Apnea. *Am J Respir Crit Care Med.* 2018;197(9):1187-1197.

2. Taranto-Montemurro L, Messineo L, Azarbarzin A, et al. Effects of the Combination of Atomoxetine and Oxybutynin on OSA Endotypic Traits. *Chest.* 2020;157(6):1626-1636.

3. Ong JS, Touyz G, Tanner S, Hillman DR, Eastwood PR, Walsh JH. Variability of human upper airway collapsibility during sleep and the influence of body posture and sleep stage. *J Sleep Res.* 2011;20(4):533-537.
